# Copper-transporting P-type adenosine triphosphatase (ATP7A) is associated with platinum-resistance in non-small cell lung cancer (NSCLC)

**DOI:** 10.1186/1479-5876-10-21

**Published:** 2012-02-03

**Authors:** Zhuang-hua Li, Miao-zhen Qiu, Zhao-lei Zeng, Hui-yan Luo, Wen-jing Wu, Feng Wang, Zhi-qiang Wang, Dong-sheng Zhang, Yu-hong Li, Rui-hua Xu

**Affiliations:** 1 State Key Laboratory of Oncology in South China, Sun Yat-Sen University Cancer Center, Guangzhou 510060, China; 2 Department of Medical Oncology, Sun Yat-Sen University Cancer Center, 651 Dong Feng Road East, Guangzhou 510060, China; 3 Department of Medical Oncology, Dongguan People's Hospital, Dongguan523059, Guangdong, China

## Abstract

**Background:**

Copper export protein ATP7A is important for maintaining copper homeostasis. Recent studies have shown that copper transporters are also involved in the transport of platinum. The goal of this study was to determine the role of ATP7A in the platinum-resistance of non-small cell lung cancer (NSCLC).

**Methods:**

Sensitivities to platinums were detected by MTT assay and drug-resistance related genes were analyzed by real-time PCR and immunoblotting between DDP-sensitive A549 and the corresponding DDP-resistant cell subline (A549/DDP). ATP7A expression was evaluated by immunohistochemistry in tumor tissues of unresectable NSCLC patients who received cisplatin-basing chemotherapy.

**Results:**

The expression of ATP7A was significantly higher in A549/DDP cell subline than in A549 cells at both mRNA and protein levels. The silencing of ATP7A expression in A549/DDP by siRNA partially reversed DDP-resistance (29.62%) and increased cell apoptosis. ATP7A expression was detected in 41.6%of NSCLC patients, but not in adjacent stroma nor normal lung tissues. ATP7A-positive patients had a significantly poorer histological grade (p = 0.039) and poorer response to platinum-basing chemotherapy (p = 0.001) compared with ATP7A-negative patients. Cox's proportional hazards analysis showed that ATP7A expression was an independent prognostic factor for overall survival (p = 0.045).

**Conclusions:**

ATP7A overexpression played an important role in platinum-resistance of NSCLC, and was a negative prognostic factor of NSCLC patients treated with platinum-based chemotherapy.

## Background

Non-small cell lung cancer(NSCLC) accounts for 80% of all lung cancers and is a leading cause of cancer-related death during the last decades. Platinum-based chemotherapy is the major treatment for advanced NSCLC. However, the best overall response rate is only 30-50% [1]. The intrinsic/acquired resistance of tumor to platinum derivates limit the effectiveness of therapy [2]. Consequently, there is great need for elucidating the mechanism of resistance to platinum in NSCLC.

There are a viariety of factors contributing to platinum-resistance, including decreased drug accumulation, enhanced detoxification, and increased DNA repair efficiency [3]. Although multidrug resistance-associated protein 2 (MRP2/ABCC2) has been reported to transport DDP and confered resistance to DDP [4], actually the mechanisms of decreasing of DDP accumulation in resistant cancer cells have not been fully elucidated yet. One of the mechanisms might be the vesicular transport depending on copper transporters.

Copper uptake protein CTR1, copper export proteins ATP7A and ATP7B are important for maintaining copper homeostasis. Recent studies have shown that copper transporters are also involved in the transport of platinum. DDP-resistant cells showed cross-resistant to copper, and vice versa [5]. Evidences also suggested that some copper influx transporters especially CTR1 were involved in the cellular uptake of DDP, CBDCA and L-OHP, and the other two copper transporters ATP7A and ATP7B import copper to golgi apparatus which regulates the efflux of DDP [6]. As a P-type ATPase copper transporter, ATP7A is expressed in many tissues except for liver and sustains not only copper-dependent tyrosinase in melanosomes [7] but also dopamine beta monooxygenase, lysl oxidase and copper containing protein. ATP7B has been reported to relate to DDP-resistance first [8,9]. Then, another copper transporter ATP7A has also been implicated in platinum-resistance later [10,11].

Nevertheless, it is generally accepted that platinum-resistance most likely has multiple mechanisms and the mechanisms of resistance may differ depending on the cell types [12]. Whether NSCLC shares common drug resistance mechanisms with other cancers or possesses its own distinct characteristics is still unclear. Hence, a better understanding of the active mechanism of platinum-resistance in NSCLC may lead to new treatment strategies and allow selection of patients for specific treatment modalities.

## Methods

### Drugs

DDP and CBDCA were purchased from Bristol-Myers Squibb (NY, USA), and L-OHP was provided by Sanofi-aventis (Paris, France). DDP and CBDCA were diluted and stored according to the method described [13]. L-OHP was diluted in water stored as a 12.6 mM stock solution at -20°C.

### Cell Lines and Cell Culture

Human NSCLC cell lines included the parental cell line A549 and the cell subline A549/DDP which was resistant to DDP. All the cells were generously provided by State Key Laboratory of Oncology in Southern China and were all cultured according to the method described [13]. Cells used for the experiments were in the logarithmic phase of growth.

### MTT Assay

The cytotoxicities of DDP, CBDCA and L-OHP were determined by MTT assay according to the method described [14]. The dose-response curve could be plotted with 50% inhibitory concentration (IC_50_). Resistance index (RI) was calculated as IC_50 _resistant/IC_50 _sensitive cell line.

### Real-time PCR

The mRNA expressions of ATP7A in A549 and A549/DDP cells were determined using real-time PCR according to the method described [15]. Briefly, total RNA from cultured cells was extracted by using the Trizol reagent (Invitrogen) according to the manufacturer described, and cDNAs were amplified and quantified in ABI Prism 7500 Sequence Detection System (Applied Biosystems) by using dye SYBR Green I (Invitrogen). The sequence was as following: ATP7A, forword: 5'-GCCTGCGTACGTGGATTTAT-3' and reverse: 5'-TCAATGGTCCAAACACAGGA-3'. GAPDH was used as an internal control (forward: 5'-ACCA-CAGTCCATGCCATCAC-3' and reverse: 5'-TCCACCAC-CCTGTTGCTGTA -3').

### Immunoblotting

The protein expressions of ATP7A and GAPDH were determined using immunoblotting according to the method described [14]. Primary antibodies were diluted in a blocking solution 1: 500 for ATP7A (Abcam, Cambrige, UK) and 1:5000 for GAPDH (Boster, Wuhan, China).

### siRNA Transfection

ATP7A siRNA sequences were targeted exon 11 (sense, GCAACUAUUGUAACUCUUG dTdT; antisense, CAAGAGUUACAAUAGUUGC dTdT) [16]. A nonsilencing siRNA sequence, shown by BLAST search which did not share sequence homology with any known human mRNA was used as control for ATP7A-targeting experiments. The siRNA sequences were synthesized by Guangzhou RiboBio (Ghuangzhou, China). The transfection of siRNA was performed using lipofectamine-2000 (Invitrogen, CA, USA) according to the manufacturer's recommendation. Oligofectamine/siRNA complexes were formed in serum free DMEM by adding siRNA (25, 50 or 100 nM final concentration) to 5 μl of Oligofectamine (Invitrogen, CA, USA) per well. Complexes were allowed to form at 25°C for 10 min and added to wells (500 μl per well). After 4 hours of transfection, the culture medium containing 10% serum was added. The assays were carried out 24, 48, 72 and 80 hours post transfection.

### Intracellular accumulation of DDP

For the characterization of DDP uptaking, 1 × 10^6 ^cells were seeded in 100 mm tissue culture dishes and transfected with siRNA_ATP7A and siRNA_Control by lipofatamine 2000, 36 hrs later exposed to 60 μM freshly prepared DDP. After 12 hrs of exposure to the drugs, the media was discarded quickly and the cells were washed three times with 5 ml ice-cold PBS buffer. Then cells were trypsinized, scraped and resuspended in fresh drug-free medium and centrifuged at 1,000 rpm for 5 mins. The supernatant was discarded and the pellet was washed twice in 1 ml ice-cold PBS buffer. After centrifugation for 2 mins at 4°C and 6,000 × g the supernatant was discarded again and the cell pellet was frozen at -20°C until analysis. Immediately after thawing, the cells were lysed with concentrated nitric acid for 20 mins in the water bath at 60°C. The lysed samples were then diluted to 3.5 ml of distilled water and intracellular platinum (Pt) concentrations were measured by the atomic spectrometer (Varian AA Duo, USA). Pt levels were expressed as μM/1 × 106 cells, with the cell number determined by counting (Nexcelom Cellometer Auto T4 cell counter, USA) in parallel cultures. Experiments were performed in duplicate and the values expressed were the means ± SD of the three independent experiments.

### Flow Cytometry

Apoptotic or necrotic cell death was determined by flow cytometric analysis of cells double stained with Annexin V-FITC and propidium iodide (PI) using an assay kit from KeyGen Biotech (Nanjing, China). A549/DDP cells were plated into 6-well plates and adhered for overnight. Then 100 nM final concentration of siRNA (targeted-ATP7A or nonsilencing siRNA) was added to wells for transfection. After 80 hours of transfection including 72 hrs for DDP incubation, cells were collected, washed with cold PBS, and suspended in binding buffer. The cells were stained with Annexin V-FITC and PI for 15 minutes at room temperature in the dark. The samples were analyzed with a flow cytometry (BECKMAN-COULTER FC500, CA, USA).

### Patients and Samples

Between October 2006 and August 2008, the medical records of pathology-proven unresectable (local advanced or distant metastasis) non-small cell lung cancer patients who were diagnosed and received cisplatin-based chemotherapy in the Cancer Center of Sun Yat-Sen University were retrospectively analyzed. We excluded patients who had a history of other primary cancers. Patients who were considered for the final analysis should have a performance status (PS) of grade 0 or 1 and didn't undergo surgery or radiotherapy, but was primarily treated with first-line cisplatin-based chemotherapy. We totally identified 89 formalin-fixed paraffin-embedded samples, corresponding to pretreatment bronchoscopic or fine-needle aspiration biopsies. Diagnosis was based on conventional morphological examination of the sections stained with H&E staining, and staging of tumors were classified according to the UICC classification. The cisplatin-based regimen included cisplatin (75-80 mg/m^2^), paclitaxel (175-225 mg/m^2^), or gemcitabine (800-1000 mg/m^2^, d1, d8) or navelbine (25-40 mg/m^2 ^d1, d8). All of the cases received 1-6 courses cisplatin containing regimens. Response to cisplatin-based chemotherapy was evaluated by physicians according to Response Evaluation Criteria in Solid Tumors (RECIST) criteria. The follow-up for all of the patients was updated in June 1, 2009 (median follow-up time was 12.5 months; range, 1.7-32 months). As of the last follow-up time, all patients had died of tumor progression. The study was approved by the institutional review board of the hospital.

### Immunohistochemical Staining

Immunohistochemical staining was performed according to the guidelines of the Catalyzed Signal Amplification System (ZSGB-BIO, Beijin, China). The slides were incubated with a 1:200 dilution of anti-ATP7A antibody (Abcam, Cambrige, UK). Adult normal kidney and lung samples were taken as positive control and negative control respectively. The slides were examined and scored by two pathologists independently without knowledge of clinical information of the patients. If more than 10% of the tumor cells were stained, the samples were considered to be ATP7A-positive carcinomas [17].

### Statistical Analysis

Data analysis was performed using SPSS 16.0 statistical software package (SPSS, IL, USA). Continuous variables were analyzed using Student's t test. As qualitative variables, the clinicopathologic characteristics of 89 patients with NSCLC were analyzed using chi-square test. Survival curves were determined using Kaplan-Meier method, and differences in survival between subgroups were compared with log-rank test. Multivariate prognostic analysis was performed using Cox proportional hazards model. All the reported P-values were two-sided. *P *< 0.05 was hypothesized to be significant.

## Results

### A549/DDP cells is resistant to platinum-derivate drugs

The drug sensitivities of A549 and A549/DDP cells to three platinum-derivate drugs (DDP, CBDCA and L-OHP) were shown in Table 1. The IC_50 _were 3.79 and 29.83 uM for DDP, 45.85 and 205.70 uM for CBDCA, 7.68 and 18.07 uM for L-OHP in A549 and A549/DDP respectively. Compared with A549 cells, A549/DDP showed 7.88 folds enhanced resistance to DDP, and both exhibited cross-resistance to CBDCA and L-OHP (4.49 and 2.35 folds respectively). RIs were the highest for DDP and the lowest for L-OHP.

**Table 1:** Drug sensitivity of A549 and A549/DDP cells to platinum derivatives (DDP, CBDCA and L-OHP)

	^a^IC_50 _(mean ± SD, uM)			
	
Drugs	A549	A549/DDP	^b^RI	*P*
DDP	3.79 ± 0.11	29.83 ± 1.25	7.88	< 0.001

CBDCA	45.85 ± 1.24	205.70 ± 3.02	4.49	< 0.001

L-OHP	7.68 ± 0.28	18.07 ± 0.39	2.35	< 0.001

Cells were precultured for 24 hrs at a density of 5*10^3 ^cells/well in 96-well multiplates.Cytotoxicity was determined 72 hrs after drug exposure by MTT. a, IC_50_, inhibitory concentration that kills 50% of cell population. Data represents the mean ± SD of at least three independent experiments performed with 8 wells at each drug concentration tested. b, RI, resistance index calculated as IC_50 _resistant/IC_50 _sensitive cell line. SD, standard deviation; DDP, cisplatin; CBDCA, carboplatin; L-OHP, oxaliplatin. *P *< 0.05 was considered as a significant change in drug sensitivity (Student's t-test)

### ATP7A was overexpressed in A549/DDP

**Figure 1:**
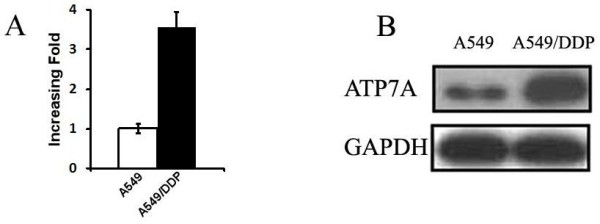
**Expression of ATP7A at RNA and protein levels in A549 and A549/DDP cells**. a, total of RNA isolated from A549 and A549/DDP cells was subjected to real-time PCR for *ATP7A *b, ATP7A protein expression was determined by Western-blot analysis using anti-ATP7A antibody. Equal loading was confirmed with GAPDH as a control in real-time PCR and Western-Blot.

The expression of ATP-binding cassette (ABC) transporters in A549 and A549/DDP cell lines were examed by real-time PCR and immunoblotting. As shown in Figure 1a, ATP7A mRNA expression in A549/DDP was much higher than in A549 (Figure 1a) and the protein expression was also comfirmed by immunoblotting. There was no significant difference in expressions of MDR1, ABCG2, MRP1, LRP, GST-pi, DNApolβ, CTR1 and ATP7B (data not shown). These results indicated that ATP7A might be related to the platinum-derivates resistance in A549/DDP cells.

### Inhibition of ATP7A expression by siRNA enhance chemosensitivity to DDP in A549/DDP

**Figure 2:**
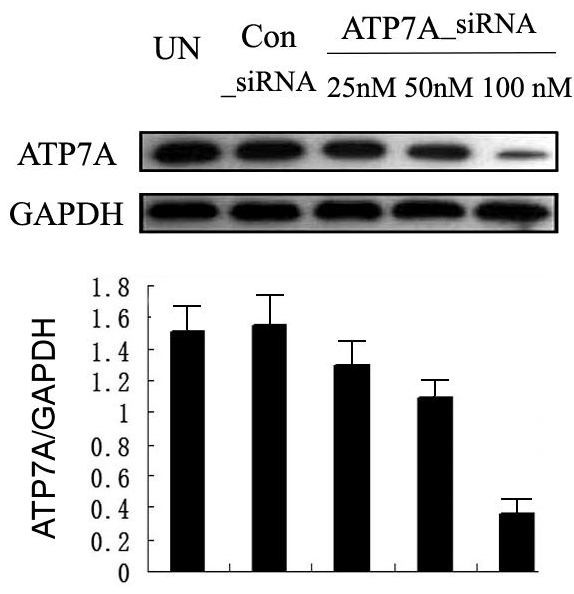
**Effect of ATP7A-siRNA 80 h on ATP7A protein expression in A549/DDP ells**. Equal loading was confirmed with GAPDH as a control. Western blot analysis of ATP7A in A549/DDP cells following treatment with control siRNA and different concentrations of targeted ATP7A siRNA (25 nM, 50 nM and 100 nM) for 80 h. Protein expression was quantified by Quantity One software. Results are the representative of three similar experiments.

To further determine the role of ATP7A in platinum-resistance of A549/DDP cells, we used RNA interference system to knockdown the expression of ATP7A in A549/DDP cells. As shown in Figure 2, 100 nM ATP7A-targeted siRNA sequences, the silenced most expression of ATP7A protein in A549/DDP cells (76.0 ± 0.08%). The reversal effect of the siRNA transfection (100 nM, 80 hrs) on the DDP-resistance of A549/DDP was investigated by MTT assay. As shown in Table 2, siRNA transfection targeting ATP7A reduced IC_50 _of A549/DDP cells significantly compared with controls (reversal effects of 29.62%). Silencing ATP7A was able to partially reverse DDP-resistance in A549/DDP cells. These results indicated that ATP7A played an important role in the modulation of platinum-resistance in A549/DDP.

**Table 2:** Partially reversal effect after ATP7A's silence by siRNA for 80 h in the DDP-resitant subline A549/DDP

cell lines	IC_50 _(mean ± SD, uM)	^a^reversal effect(%)	*P*
A549/DDP	29.33 ± 0.20		

A549/DDP Control SiRNA	29.51 ± 0.17		> 0.05

A549/DDP ATP7A SiRNA	20.77 ± 0.28	29.62	< 0.001

ATP7A siRNA group is A549/DDP cells treated with ATP7A siRNA sequences at the final 100 nM concentration for 8 hrs and then added DDP at various concentrations for 72 hrs. Control siRNA group is A549/DDP cells treated with nonsilencing siRNA sequences at the final 100 nM concentration for 8 hrs and then added DDP at various concentrations for 72 hrs. a, reversal effect was determined by the percentage of the IC_50 _difference between Control siRNA group and ATP7A siRNA group to the IC_50 _of Control SiRNA groups. Data represents the mean ± SD of at least three independent experiments performed with 8 wells at each drug concentration tested. *P *< 0.05 was considered as a significant change in drug sensitivity (Student's t-test). SD, standard deviation; DDP, cisplatin

**Figure 3:**
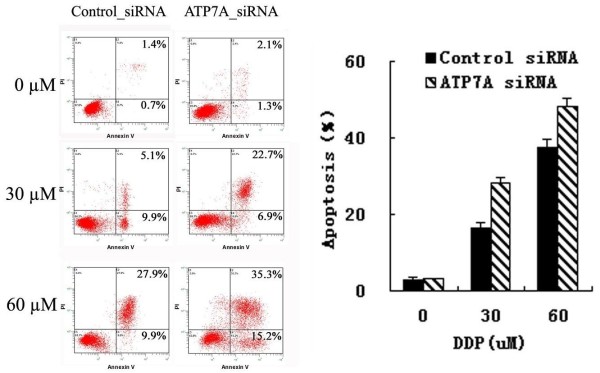
**Increasing DDP induced apoptosis after ATP7A's silence for 80 h by siRNA in A549/DDP cells**. Apoptotic or necrotic cell death was tested by flow cytometric analysis. Control siRNA, added 100 nM nonsilencing siRNA sequences for 8 h and then added DDP for 72 h; ATP7A siRNA, added 100 nM SiRNA targeting ATP7A for 8 h and then added DDP for 72 h. Columns, mean of three independent experiments; bars, SD.

To further determine whether ATP7A-targeted siRNA transfection could reverse the platinum-resistance of A549/DDP, DDP-induced apoptosis was detected by Annexin V-PI assay. As shown in Figure 3, without DDP, there was little apoptosis and no significant difference between ATP7A-targeted siRNA group and control siRNA group. But after treatment with different concentrations of DDP for 72 hrs, cell apoptotic rates in ATP7A siRNA group were significantly higher than in control siRNA group (28.37 ± 1.25% compared to 16.43 ± 1.40% at 30 uM DDP and 48.33 ± 2.16% compared to 37.43 ± 2.17 at 60 uM DDP). Thus, silence of ATP7A by siRNA enhanced DDP-induced apoptosis significantly compared with control siRNA at different DDP concentrations (*P *< 0.01). These results 'further confirmed that ATP7A played a role in modulation of platinum-resistance in A549/DDP cells.

### Effect of ATP7A on the intracellular accumulation and efflux of platinum

To investigate how does ATP7A modulated platinum resistance, cellular accumulation of DDP was examined in A549, A549/DDP and A549/DDP cells transfected with siRNA ATP7A or siRNA control. Cells were incubated in medium containing 60 μM freshly prepared DDP for 12 hrs and the level of DDP in cell lysates was then measured. As shown in Table 3, intracellular amounts of DDP in A549/DDP cells transfected with siRNA control (0.06 ± 0.005 uM/10^6 ^cells) were much lower than in the parental A549 cells (0.69 ± 0.007 uM/10^6 ^cells) (*p *< 0.001). and intracellular amounts of DDP in A549/DDP cells transfected with siRNA ATP7A (0.13 ± 0.003 uM/10^6 ^cells) were significantly higher than in A549/DDP cells transfected with siRNA control (*p *< 0.001). These data strongly suggested that the decreased DDP accumulation in A549/DDP cells was due to an ATP-dependent DDP transporting activity of ATP7A, which mediated platinum-resistance.

**Table 3:** Intracellular accumulation of DDP in A549 cells, A549/DDP cells and A549/DDP cells treated with control siRNA or ATP7A siRNA respectively

cells	Concentration (μmol/10^6 ^cells)	*P*
A549	0.69 ± 0.007	

A549/DDP	0.06 ± 0.002	< 0.001

A549/DDP_Control_siRNA	0.06 ± 0.005	

A549/DDP_ATP7A_siRNA	0.13 ± 0.003	< 0.001

Pt levels were expressed as μmol/1 × 10^6 ^cells (A549 cells, A549/DDP cells and A549/DDP cells treated with control siRNA ATP7A siRNA). Experiments were performed in duplicate and the values expressed were the means ± SD of the three independent experiments

### ATP7A expression was negatively correlated with response to DDP-based chemotherapy in NSCLC

**Figure 4:**
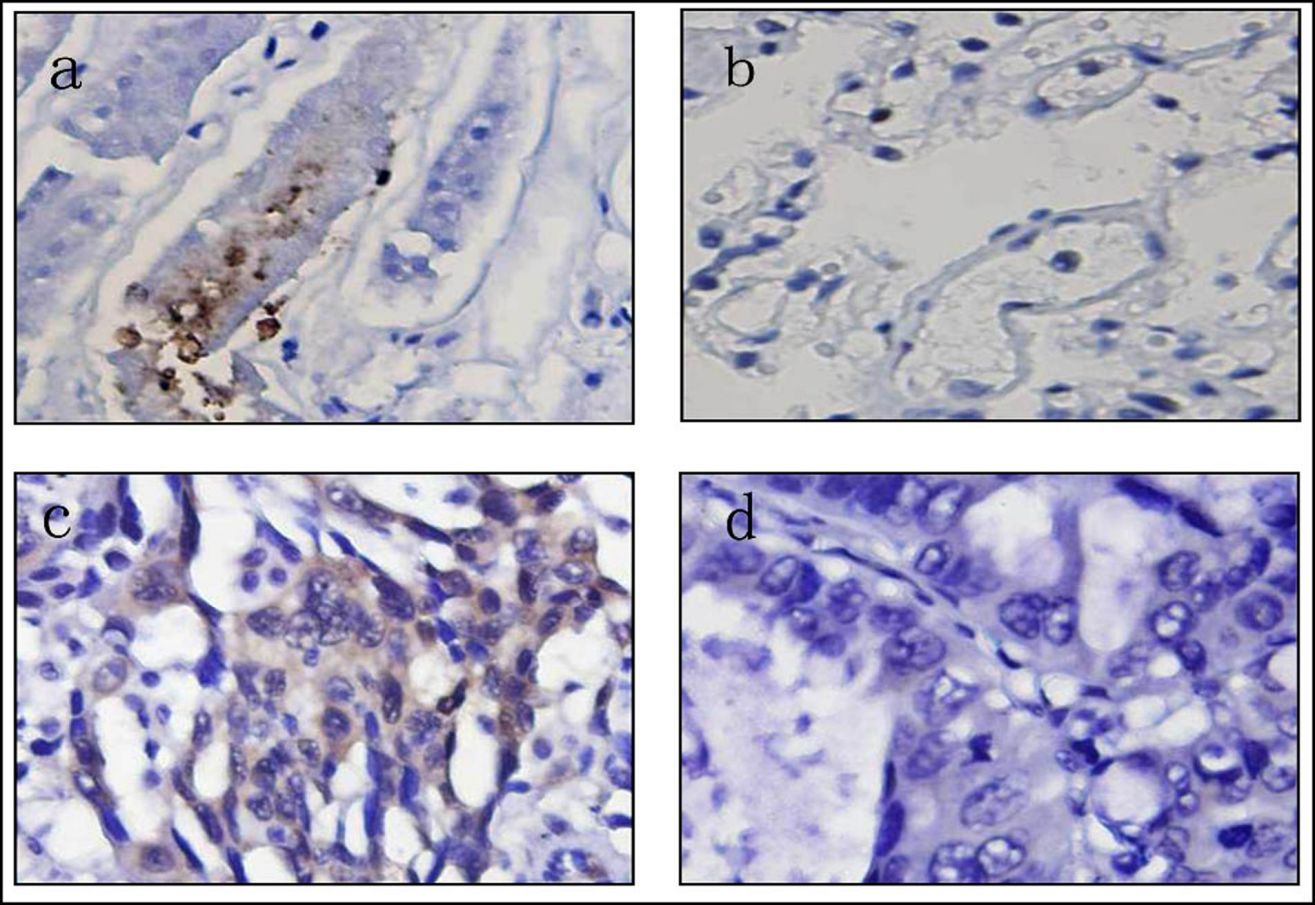
**Immunohistochemical staining of normal and NSCLC human tissues for ATP7A (magnification, ×400)**. a, adult normal kidney tissue as positive control; b, adult normal lung tissue as negative Control; c, ATP7A high positive case, almost all of tumor cells showed strong positive reaction in the cytoplasm; d, ATP7A negative case, ATP7A expression was not detectable in the cytoplasm.

**Figure 5:**
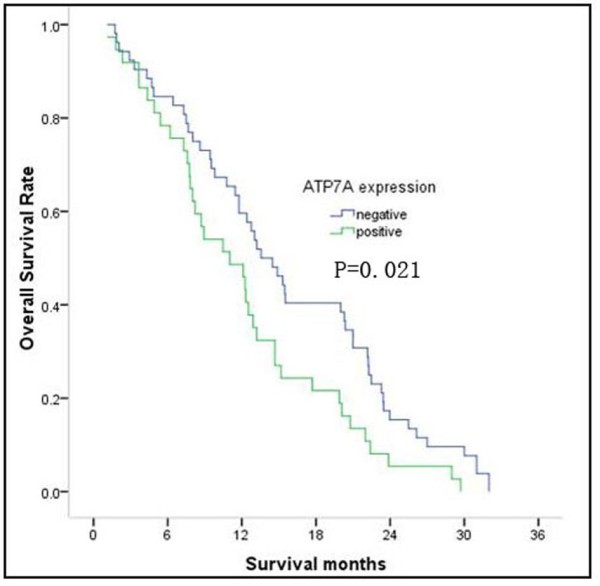
**Overall survival curves of NSCLC treated with platinum-based chemotherapy**. Comparison of survival curves for patients whose tumors stained positive for ATP7A with those patients whose tumors were classified as negative for ATP7A expression. Curves present the results for all patients. P value was conducted by Log-rank test.

To investigate the expression of ATP7A in NSCLC tissue, and analyze its correlation to the clinicopathologic features and prognosis of NSCLC, immunohistochemistry method were employed. As shown in Figure 4, ATP7A was detected in cytoplasmic staining of tumor cells in 37 of 89 (41.6%) tumors but not in adjacent stroma nor normal lung tissue. Associations were sought between ATP7A expression and the clinicopathologic characteristics of 89 patients including age, gender, stage, performance status (PS), histological subtype, histological grade, serum Carcinoembryonic Antigen (CEA) status, response to chemotherapy and overall survival (OS). The clinical parameters of the 89 patients in this study were summarized in Table 4. ATP7A expression was negatively correlated with response to DDP-basing chemotherapy (*P *= 0.001) and histological grade (P = 0.039). No significant association was found between ATP7A expression and age (*P *= 0.469), gender (*P *= 0.442), stage (*P *= 0.436), PS (*P *= 0.361), histological subtype (*P *= 0.67) and serum CEA status (*P *= 0.661). The median overall survival (mOS) of all patients was 12.53 months. In 37 patients with ATP7A positive staining, the mOS was 11.03 months, and the mOS was 14.02 months in 52 patients with ATP7A negative staining. As shown in Figure 5, Kaplan-Meier analysis indicated that ATP7A positive patients had inferior survival compared with ATP7A negative patients (*P *= 0.021, log-rank test). In univariate analysis, ATP7A, stage, PS, serum CEA status and histological grade were significantly correlated with survival (*P *= 0.021, 0.019, 0.004, 0.022 and 0.013 respectively). Cox's proportional hazards model analysis showed that 4 independent factors significantly related to overall survival: ATP7A (RR = 1.570, 1.010-2.440, *P *= 0.045), stage (RR = 1.759, 1.256-2.464, *P *= 0.001), CEA status (RR = 1.828, 1.162-2.874, *P *= 0.009) and histological grade (RR = 1.704, 1.205-2.408, *P *= 0.003).

**Table 4:** The clinicopathologic characteristics of 89 patients with NSCLC treated with platinum-based chemotherapy only in this study

Characteristics	Number of patients			
	
	Total	ATP7A(-)	ATP7A(+)	*P*
Age				

median	57	57	57	

range	31-79	35-79	31-77	0.469

Gender				

male	64	39	25	

female	25	13	12	0.442

Response to chemo				

PR	29	24	5	

SD+PD	60	28	32	0.001

Stage				

III	48	28	20	

IV	41	24	17	0.436

PS				

0	46	29	17	

> 0	43	23	20	0.361

Histology				

squamous	25	14	11	

adenocarcinoma	64	39	25	0.67

Grade				

low	48	28	20	

moderate	27	12	15	

high	14	12	2	0.039

CEA status				

normal	34	18	16	

elevated	55	34	21	0.661

## Discussion

This article sheds new light on the potential function of copper transporter ATP7A in NSCLC. In our study, as expected, DDP-resistant cell subline (A549/DDP) was much more (7.88 folds) resistant to DDP than the parental cells (A549). Note that this cell line also exhibited cross-resistance to CBDCA (4.49 folds) and L-OHP (2.35 folds). The resistant indexes of L-OHP (2.352) was much lower than that of DDP (7.88). That could be caused by the chemical structure of L-OHP. L-OHP bears a 1,2-diaminocyclohexane (DACH) ligand which is quite different from DDP and CBDCA, conferring it a markedly different spectrum of activity from DDP and CBDCA [18]. A549/DDP was exposed DDP by pulse treatment which is similar to the clinical pulse protocol for NSCLC patients to select for the drug-resistant subline. CBDCA and L-OHP were not exposed to A549/DDP in the process of establishing the cell subline. We found that ATP7A expression correlated with the resistance to DDP, then might at a lesser extent to CBDCA and L-OHP.

Reduced drug accumulation has been related to ATP-binding cassette (ABC) transporters. Overexpression of MDR1(Pgp) was capable of pumping out a variety of diverse chemotherapy drugs to reduce intracellular drug concentration and multidrug resistance (MDR) [19], and A link was identified between MDR transporter Pgp, and MAPK signaling and invasion in human melanoma cells [20]. In clinical, MDR1 expression in childhood ALL is an independent adverse prognostic factor on outcome, a useful biological marker of response in these patients [21]. Recently, a report found that single nucleotide polymorphisms of ABCC5 and ABCG1 transporter genes correlated to irinotecan-associated gastrointestinal toxicity in colorectal cancer patients [22]. But in our study, the mRNA expression levels of MDR1, ABCG2, MRP1, LRP, GST-pi, DNApolβ, CTR1 and ATP7B were almost the same between A549/DDP and A549 cells, suggesting that the platinum-resistance in A549/DDP cells is independent of ABC transporters. However, ATP7A expression in both mRNA and protein levels significantly increased in A549/DDP cells comparing with the parental cells respectively. Furthermore, in A549/DDP cells, ATP7A siRNA knockdown (76.0 ± 0.08%) was able to partially reverse DDP-resistance (29.62%). The cytotoxicity of platinum-compounds was thought to be determined primarily by their DNA adducts [23], which then induced cell apoptosis [24]. In our study, ATP7A silencing significantly increased cells apoptosis rates in A549/DDP cells. Thus, the reduced expression of ATP7A achieved by siRNA knockdown resulted in enhancement of DDP-sensitivity and increased DDP-induced apoptosis. But there should be many factors related to the resistance to DDP in A549/DDP cells, but not only ATP7A. So it isn't surprising that knocking down ATP7A exepression in A549/DDP cells only partially reversed the resistance to DDP but not mostly.

The intracellular amounts of DDP in A549/DDP cells was much lower than in the parental A549 cells, suggesting the platinum resistance of A549/DDP is due to decreased drug accumulation. SiRNA-knockdown ATP7A in A549/DDP cells increased the intracellular amounts of DDP. These results indicate that the platinum-resistance in A549/DDP cells is mediated by decreasing drug accumulation of ATP7A. Overexpression of ATP7A in A549/DDP cells might pump more platinum into trans-Golgi network, thus decrease cellular platinum concentration and keep them from accessing their key cytotoxic targets in the nucleus, resulting in DDP-resistance. Recently the copper transporters have been demonstrated to be involved in platinum-resistance in some cancers. Samimi G demonstrated that overexpression of ATP7A in human ovarian carcinoma cells confered resistance to DDP, CBDCA and L-OHP by sequestering platinum analogues in intracellular compartments and preventing their reaction with nuclear DNA [10]. Plasencia C also reported that ATP7A overexpression in L-OHP resistant colorectal cancer cells [25]. Consistent with those reports, our results demonstrated that the platinum resistance is associated with ATP7A in lung cancer cell. To our knowledge, this is the first report about ATP7A related to platinum-resistance in NSCLC.

In clinical, we found that 41.6% (37/89) of NSCLC patients aberrantly expressed ATP7A. However, ATP7A protein was not detected in tumor adjacent stroma nor normal lung tissue. This suggested that ATP7A might be involved in transformation of a normal differentiated cell to a malignant tumor cell. Compared with the ATP7A-negative patients, ATP7A-positive patients had a inferiorly histological grade (*P *= 0.039), inferior response to platinum-basing chemotherapy (*P *= 0.001) and poorer OS (*P *= 0.021). Cox's proportional hazards model analysis showed that ATP7A expression was an independently prognostic factor of survival. Some studies in ovarian cancer showed similar results that increased expression of ATP7A mediated resistance to platinum derivatives in cancer cells and was associated with poor survival in ovarian cancer patients during platinum drug-based treatments [10,11].

## Conclusions

Our results demostrated that overexpression of copper efflux transporter ATP7A was responsible for platinum resistance in A549/DDP cells. These results indicated that ATP7A expression assessed by immunohistochemistry could be a chemo-resistant marker and a negative prognostic factor for survival in NSCLC patients treated with platinum-based chemotherapy, providing a basis for a better utilization of platinum-based antitumor agents and thereby improving the survival of NSCLC patients.

## Abbreviations

ATP7A: Adenosine triphosphatase; NSCLC: Non-small cell lung cancer; RECIST: Response Evaluation Criteria in Solid Tumors; ABC: ATP-binding cassette; MDR: Multidrug resistance; MRP1: Multidrug resistance-associated protein 1; LRP: Lung resistance protein; ABCG2: ATP-binding cassette sub-family G member 2 protein; GST: GSH-S-transferase; DNA polβ: DNA polymerase beta; PI: Propidium iodide; PS: Performance status; OS: Overall survival. RT-PCR: Reverse Transcriptase-Polymerase Chain Reaction; CEA: Carcinoembryonic Antigen.

## Competing interests

We have no financial or personal relationships with other people or organizations that would bias our work. No benefits in any form have been received or will be received from a commercial party related directly or indirectly to the subject of our article.

## Authors' contributions

LZH and QMZ carried out the real-time PCR, participated in the clinical data collecting of the gastric carcinoma patients and drafted the manuscript. ZZL carried out the MTT and immunoblotting. LHY participated in the blood sample collecting. WWJ carried out the flow cytometry. WF performed the statistical analysis. WZQ and ZDS participated in the design of the study. LYH drafted the manuscript and participated in the statistical analysis. XRH conceived of the study, and participated in its design and coordination and helped to draft the manuscript. All authors read and approved the final manuscript.
